# Classical Swine Fever Virus vs. Classical Swine Fever Virus: The Superinfection Exclusion Phenomenon in Experimentally Infected Wild Boar

**DOI:** 10.1371/journal.pone.0149469

**Published:** 2016-02-26

**Authors:** Sara Muñoz-González, Marta Pérez-Simó, Andreu Colom-Cadena, Oscar Cabezón, José Alejandro Bohórquez, Rosa Rosell, Lester Josué Pérez, Ignasi Marco, Santiago Lavín, Mariano Domingo, Llilianne Ganges

**Affiliations:** 1 IRTA, Centre de Recerca en Sanitat Animal (CReSA, IRTA-UAB), Campus de la Universitat Autònoma de Barcelona, 08193 Bellaterra, Spain; 2 Servei d'Ecopatologia de Fauna Salvatge, Departament de Medicina i Cirurgia Animals, Universitat Autònoma de Barcelona, 08193 Bellaterra, Spain; 3 Departament d’Agricultura, Ramaderia, Pesca, Alimentació i Medi natural, Generalitat de Catalunya, 08007 Barcelona, Spain; 4 Centro Nacional de Sanidad Agropecuaria (CENSA), La Habana, Cuba; 5 Departament de Sanitat i Anatomia Animals (DAAM), Universitat Autònoma de Barcelona, 08193 Bellaterra, Barcelona, Spain; Thomas Jefferson University, UNITED STATES

## Abstract

Two groups with three wild boars each were used: Group A (animals 1 to 3) served as the control, and Group B (animals 4 to 6) was postnatally persistently infected with the Cat01 strain of CSFV (primary virus). The animals, six weeks old and clinically healthy, were inoculated with the virulent strain Margarita (secondary virus). For exclusive detection of the Margarita strain, a specific qRT-PCR assay was designed, which proved not to have cross-reactivity with the Cat01 strain. The wild boars persistently infected with CSFV were protected from superinfection by the virulent CSFV Margarita strain, as evidenced by the absence of clinical signs and the absence of Margarita RNA detection in serum, swabs and tissue samples. Additionally, in PBMCs, a well-known target for CSFV viral replication, only the primary infecting virus RNA (Cat01 strain) could be detected, even after the isolation in ST cells, demonstrating SIE at the tissue level *in vivo*. Furthermore, the data analysis of the Margarita qRT-PCR, by means of calculated ΔCt values, supported that PBMCs from persistently infected animals were substantially protected from superinfection after *in vitro* inoculation with the Margarita virus strain, while this virus was able to infect naive PBMCs efficiently. In parallel, IFN-α values were undetectable in the sera from animals in Group B after inoculation with the CSFV Margarita strain. Furthermore, these animals were unable to elicit adaptive humoral (no E2-specific or neutralising antibodies) or cellular immune responses (in terms of IFN-γ-producing cells) after inoculation with the second virus. Finally, a sequence analysis could not detect CSFV Margarita RNA in the samples tested from Group B. Our results suggested that the SIE phenomenon might be involved in the evolution and phylogeny of the virus, as well as in CSFV control by vaccination. To the best of our knowledge, this study was one of the first showing efficient suppression of superinfection in animals, especially in the absence of IFN-α, which might be associated with the lack of innate immune mechanisms.

## 1. Introduction

Members of the Pestivirus genus, within the *Flaviviridae family*, account for a variety of diseases in farm animals, the most economically important of which are bovine viral diarrhoea virus (BVDV) and classical swine fever virus (CSFV). Classical swine fever virus (CSFV) is the etiological agent of a highly contagious viral disease of swine affecting domestic pigs and wild boars [1], which has caused major losses in stock farming [2, 3]. CSFV is composed of a lipid envelope, a capsid and a single plus-strand RNA genome carrying a single, large open reading frame (ORF) flanked by two untranslated regions (UTRs). The ORF encodes a polyprotein of approximately 3900 amino acids, which are processed by cellular and viral proteases in the four structural proteins—C, E^rns^, E1, E2—and in the 8 non-structural proteins—N^pro^, P7, NS2, NS3, NS4A, NS4B, NS5A, and NS5B [4].

Recently, it was proved that CSFV can generate postnatal persistence by infecting both newborn piglets and wild boars with either low- and/or moderate-virulence strains, respectively. Over the six weeks after postnatal infection, most of the infected animals remained clinically healthy, despite persistent high virus titres in the blood, organs and body secretions. Importantly, these animals were unable to mount any detectable humoral or cellular immune responses. At necropsy, the most prominent gross pathological lesion was severe thymus atrophy. Four weeks after infection, PBMCs from persistently infected seronegative piglets were unresponsive to both specific CSFV and non-specific PHA stimulation in terms of IFN-γ-producing cells. These results suggested the development of an immunosuppression state in these postnatally persistently infected pigs [5, 6]. In addition, it was shown that six-week-old, persistently CSFV-infected pigs were unable to elicit specific immune responses following vaccination with a CSFV lapinised C-strain vaccine (HCLV) [7]. Interestingly, the RNA of the vaccinal C-strain was undetectable by specific RT-PCR [8] in any of the samples analysed after vaccination, including blood, nasal and rectal swabs, or organs throughout the experiment, suggesting a phenomenon of homologous interference, also known as superinfection exclusion (SIE), between the high viral load generated by the primary persistent infection and the CSFV vaccine strain.

The SIE phenomenon, defined as the ability of a primary virus infection to interfere with a secondary infection by the same or a closely related virus, has been described in a broad range of virus-host systems, including bacteria, plants, and animals, and in important pathogens of humans, such as rubella virus, human immunodeficiency virus (HIV), and hepatitis C virus (HCV), among others [9–20]. From an evolutionary standpoint, SIE might be a conservative strategy, reducing the likelihood of recombination events between related strains [17, 21, 22], thus determining the stability of viral sequences within the same cell. From a practical standpoint, SIE has significant implications for the treatment or prevention of viral infections. In this regard, cross-protection of crops by purposeful infection with milder virus isolates is a widely accepted practice, and it is viewed as an effective and economical antiviral management strategy [23]. Additionally, transplantation of HCV-infected liver grafts has been suggested as a treatment for already infected patients, given that the transplantation of a healthy organ would lead to rapid damage to the newly transplanted liver by the virus of the recipient patient [15, 24].

Previous studies conducted in cell cultures with BVDV demonstrated that cells acutely infected with this virus were protected from a second infection by a homologous BVDV strain [17]. Additionally, it was shown that CSFV is generally noncytopathic, and it readily establishes persistent infections in cell culture. Nevertheless, when persistently infected cultures were serially passaged more than 100 times, spontaneous generation of cytopathogenic (cp) CSFV variants could occur. The few surviving cells of the cytopathic effect (CPE), although still infected, were also protected from the CPE after superinfection with cp CSFV [25]. Both studies supported the ability of pestiviruses to generate SIE in cell cultures. Thus, along with the availability of a persistent infection model of CSFV, in the present study, we sought to assess SIE against a highly virulent CSFV strain at the organism level in six-week-old wild boars, rendered persistently CSFV-infected at birth. Our results showed that SIE could occur at the systemic level in CSFV-infected swine.

## 2. Materials and Methods

### 2.1. Cells and viruses

PK-15 cells (ATCC CCL 33) and SK6 cells [26] were cultured in Dulbecco's Modified Eagle Medium (DMEM), supplemented with 10% foetal bovine serum (FBS), Pestivirus-free, at 37°C in 5% CO_2_. The cells were infected with 0.1 TCID_50_/cell in 2% FBS, and the virus was harvested 48 h later. Additionally, ST cells (ATCC CRL 1746) were cultured in DMEM, supplemented with L-glutamine (2%) and 10% foetal bovine serum (FBS), Pestivirus-free at 37°C in 5% CO_2._ Peroxidase-linked assay (PLA) [27] was used for viral titration following the statistical methods described by Reed and Muench [28].

The Catalonia 01 (Cat01) strain used in this study was isolated from the Spanish CSF epizootic in 2000–2001 [29]. This isolate belongs to the CSFV 2.3 genogroup [30]. The course of infection by this strain was found to be mild [29, 31]. Finally, the virulent Margarita strain, which belongs to the CSFV 1.4 genogroup [29, 32, 33], was used.

### 2.2. Experimental design

To elucidate the capacity of CSFV to generate SIE, two groups (A and B), with three male, six-week-old wild boars in each, were used. These animals were acquired from Gestion Cinegetica Integral SL farm (Segovia, Spain) and were housed in the experimental isolation facilities in the biosecurity level 3 laboratory of the Centre de Recerca en Sanitat Animal (CReSA); they were fed a conventional piglet starter diet and pellets until the end of the trial (Startrite 100, Kwikstart, and Prestarter; SCA Iberica S.A., Zaragoza, Spain) and were handled according to previous studies conducted in CReSA [6]. Group A (animals 1 to 3) was used as controls, and they tested Pestivirus-free at the beginning of the study. The second group (Group B), housed in an independent isolation unit at the BSL-3 facility of CReSA, (animals 4 to 6), were postnatally persistently CSFV-infected animals. These animals, which had been intranasally infected in the first 24 h after birth with the CSFV Cat01 strain, were viraemic and apparently healthy at six weeks old, although being immunosuppressed, they lacked CSFV-specific cellular and humoral responses [5, 6]. Both groups had an average weight of 6 kg per animal. After a five-day acclimation period, all of the animals were experimentally infected by i.m. injection in the neck [33–35] with 10^5^ TCID_50_ CSFV Margarita strain. In previous studies, this viral dose caused acute CSF and often induced death at 10–15 days post-infection (dpi) [36]. Sera and nasal and rectal swabs were collected at 0, 3, 7, 10 and 13 dpi. Blood samples for the isolation of PBMCs were obtained at day 0 and at the time of euthanasia.

A trained veterinarian recorded the clinical signs daily in a blinded manner [36]. The clinical signs compatible with CSFV infection were anorexia, fever, conjunctivitis, diarrhoea, constipation, cyanosis of the skin, abdominal petechiae, dyspnoea, tremors, locomotive disturbances, reluctant walking, swaying movement of the hindquarters, posterior paresis, convulsions from mild to severe and prostration. Particular stress was placed upon the registration of nervous symptoms [29, 33, 34, 36]. The clinical status of the animals was scored from 0 to 6 [29, 33, 34, 36] as follows: 0: no signs; 1: mild pyrexia; 2: pyrexia plus mild clinical signs; 3: mild-to-moderate clinical signs; 4: moderate clinical signs; 5: moderate-to-severe clinical signs; and 6: death. For ethical reasons, the animals were euthanised when the clinical score reached 5, when exhibiting a fall of the hindquarters, when there was inability to drink or feed, when prostration occurred or when exhibiting moderate nervous disorders. After euthanasia, an exhaustive necropsy was conducted, in which the presence of pathological symptoms in different organs and tissues was evaluated. Surviving wild boars were euthanised at 13 dpi, and urine and tissues (spleen, liver, intestine, mesenteric lymph node, prescapular lymph node, bone marrow, medulla oblongata, lung, kidney, thymus and tonsil) were obtained at necropsy. Euthanasia was performed according to European Directive 2010/63/EU, using a pentobarbital overdose of 60–100 mg/kg administered via the anterior vena cava. The animal care and procedures were in accordance with the guidelines of the Good Experimental Practices (GEP), under the supervision of the Ethical and Animal Welfare Committee of the Autonomous University of Barcelona (UAB), and they were approved under number 8804, according to the existing national and European regulations. Additionally, the biosafety level of the viruses used in this study was stated as biosecurity level 3, as approved by the Biosafety Committee of the UAB, with registration assignment AR-296-15.

### 2.3. Design and validation of a new qRT-PCR for the detection of specific CSFV Margarita strain RNA

Fifteen representative sequences of the three CSFV genogroups were retrieved from GenBank and aligned using BioEdit [37]. Two primers and probes were designed for specific detection of the Margarita strain sequence (1.4 CSFV genogroup) by targeting the 5´ end of the E2 gene, as follows: forward primer (2333–2356), 5´-AAGATTACGACCACAATTTACAAC-3´; reverse primer (2411–2431), 5´-TCC TACTGACCACATTAAGCG-3´ and probe (2369–2389), 5´-CCATCAAGGCTATCTGCACGG-3´. The nucleotide positions were based on the genome sequence of the Margarita strain (GenBank accession number AJ704817). The probe was labelled with 6-FAM at the 5´ end and with BHQ1 at the 3´ end. The primers and probe were purified by reverse phase HPLC. The one-step RT-PCR protocol was undertaken using the commercially available TaqMan® One-Step RT-PCR Master Mix Reagents Kit (Applied Biosystems Roche). The real-time RT-PCR assay was optimised using a total volume of 25 μl. Real-time qRT-PCR was performed using an Applied Biosystems® 7500 Fast Real-Time PCR System. The temperature profile was 30 min at 50°C (reverse transcription), 15 min at 95°C (inactivation reverse transcriptase/activation Taq polymerase), followed by 42 cycles of 15 s at 94°C (denaturation), 30 s at 57°C (annealing) and 30 s at 68°C (elongation). Identical temperature profiles were used for all of the real-time RT-PCR runs, and fluorescence values were collected during the annealing step. Twenty CSFV RNA preparations strains were used to determine the specificity and sensitivity of the assay (Table 1) [30, 38]. To exclude the possibility of presence of CSFV Cat01 strain RNA interfering with the assay sensitivity for the CSFV Margarita strain RNA detection, mixtures from serial RNA dilutions from both viral strains were analysed. In addition, mixtures from RNA serum samples of group B (prior to the Margarita strain inoculation), with samples from group A at 7 days post-infection with the Margarita strain, were analysed.

**Table 1:** Viruses used in the standardisation of Margarita strain real-time TaqMan assay.

CSFV Genotype/subtype	References strain/isolate	Source
Genotype 1.1	HCLV vaccine (C-strain) (Muñoz-Gonzalez et al., 2015)	CReSA, Sapin
Genotype 1.4	Margarita	CReSA, Spain
Genotype 2.1	Paderborn (CSFV277 reference strain)	CReSA, Spain
Genotype 2.2	Clinical samples from experimentally infected pigs with CSF0018 reference strain (5 samples)	EU Reference Laboratory for CSF, Germany
	CSF573 reference strain (*Italy Parna’98*)	CReSA, Spain
Genotype 2.3	Clinical samples from experimentally infected pigs with CSF0864 reference strain (4 samples)	EU Reference Laboratory for CSF, Germany
	Clinical samples from experimentally infected pigs with CSF0634 reference strain (5 samples)	EU Reference Laboratory for CSF, Germany
	Uelzen (CFS639 reference strain)	CReSA, Spain
	Catalonia 01 (Pérez et al., 2012)	CReSA, Spain
	Spreda (CSF123 reference strain)	CReSA, Spain

### 2.4. Detection of CSFV RNA

RNA was extracted from all of the samples using the NucleoSpin RNA isolation kit (Macherey-Nagel), according to the manufacturer's instructions. In all cases, RNA was extracted from an initial sample volume of 150 μL to obtain a final volume of 50 μL of RNA, which was stored at -80°C. The presence of CSFV RNA in the serum and in nasal and rectal swabs, as well as in tissue samples, was analysed by a generic CSFV qRT-PCR [39]. This test was used in our laboratory for inter-laboratory comparisons of CSFV diagnoses, organised by the EU Reference Laboratory. Positive results were considered for threshold cycle values (Ct) equal to or less than 42. Samples in which fluorescence was undetectable were considered negative. Additionally, the qRT-PCR specific for the Margarita strain, designed in this work (described above), was used to distinguish those samples infected with the Margarita strain.

### 2.5. Detection of E2-specific and neutralising antibodies

Serum samples were tested with neutralisation peroxidase-linked assay (NPLA) [40], and the titres were expressed as the reciprocal dilution of serum that neutralised 100 TCID_50_ of the Cat01 or Margarita strain in 50% of the culture replicates. The detection of E2-specific antibodies was performed using a commercial ELISA kit (IDEXX); the samples were considered positive when the blocking percentage was ≥40%, following the manufacturer's recommendations.

### 2.6. Detection of IFN-α in serum samples

Anti-IFN-α monoclonal antibodies (K9 and K17) and IFN-α recombinant protein (PBL Biomedical Laboratories, Piscataway, New Jersey, USA) were used in ELISA to detect IFN-α in serum samples at 0, 3, 7 and 10 dpi [34, 41–43]. The cut-off value of the assay was calculated as the average of the optical density of negative controls (blank and negative sera before CSFV infection) plus three standard deviations. Cytokine concentrations in serum were determined using a regression line built with the optical densities of the cytokine standards used in the tests.

### 2.7. PBMCs and ELISPOT assay for CSFV-specific IFN-γ-producing cells

ELISPOT assay to detect CSFV-specific IFN-γ cells was performed as previously described [34], using PBMCs that were obtained at day 0 and at the time of euthanasia. Briefly, plates (Costar 3590, Corning) were coated overnight with 5 μg/ml capture antibody (P2G10, Pharmigen). Detection was performed using a biotinylated antibody (P2C11, Pharmigen). A total of 5x10^5^ PBMCs/well were plated in triplicate at 0.1 multiplicity of infection (MOI) of the Cat01 and Margarita CSFV strains. Moreover, the same samples were incubated in the presence of phytohaemagglutinin (PHA) (10 μg/ml). The controls were incubated in the presence of mock-stimulated wells. The numbers of spots in the media for mock-stimulated wells were considered to constitute the baseline for the calculation of antigen-specific frequencies of IFN-γ-producing cells.

### 2.8. Cell culture assay

**Fig 1:**
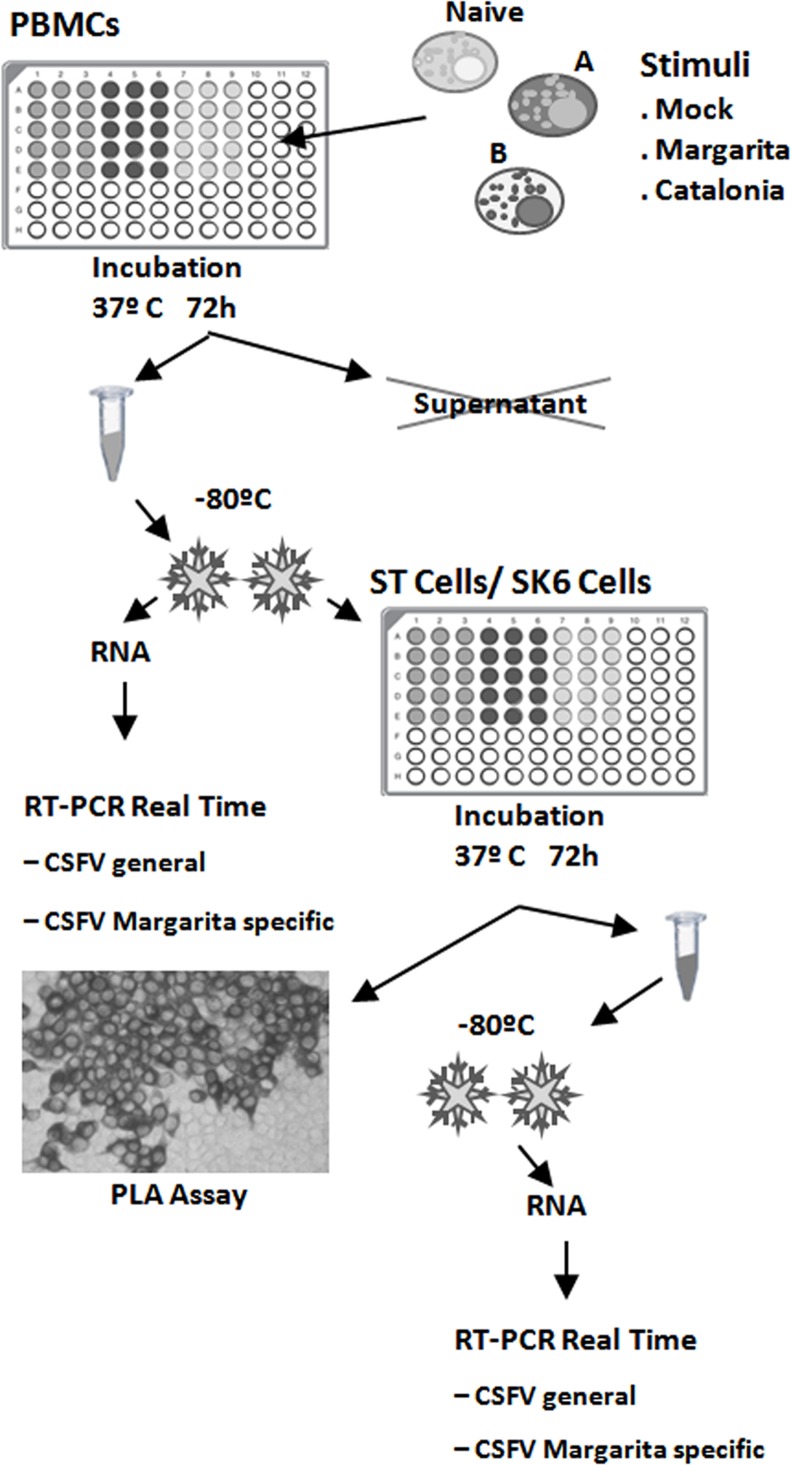
Experimental procedures to examine superinfection exclusion in PBMCs and ST cells.

Samples from animal 1 (Group A: Margarita acutely infected wild boar; 10 dpi), animal 5 (Group B: Cat01 persistently infected wild boar and superinfected with CSFV Margarita strain; 13 dpi), and a Pestivirus-free wild boar (animal 1 before infection), were used to assess SIE in PBMCs (Fig 1). The PBMCs were isolated from whole blood by centrifugation on Ficoll gradients (Histopaque-1077; Sigma). The number and viability of the PBMCs were determined by staining with Trypan blue [33]. A total of 4x10^5^ PBMCs/well from each animal were plated in quintuplicate at 37°C in 96-well plates with: (i) vehicle; (ii) the Cat01 strain at a 0.1 multiplicity of infection (MOI); and (iii) the Margarita strain (0.1 MOI). After 72 h, the PBMCs were accurately washed twice and were resuspended in a final volume of 200 μl of PBS per well. To release the virus from the cells, two freeze-thaw cycles at -80°C were undertaken, and the quintupled samples were harvested in a single aliquot. The presence of virus RNA in PBMC samples was analysed by generic CSFV qRT-PCR [39] and for the specific Margarita strain by qRT-PCR detection assay (see above, section 2.3). For virus isolation, an established cell line sensitive for specific CSFV proliferation, ST cells, were cultured at 37°C in 96-well plates in triplicate in the presence of each of the collected cell suspensions. After 72 h, the supernatants were removed, and the collected ST cells were washed twice and resuspended in 200 μl of sterile PBS. After two cycles of freeze-thaw at -80°C, the presence of CSFV RNA in the ST cell samples was analysed by qRT-PCR for CSFV [39] and the Margarita strain (see above). In parallel, a ST plate similarly inoculated with cell suspensions was used for confirmation by PLA [27]. A delta Ct (ΔCt) for Margarita strain RNA detection was calculated as the differences between (i) the Margarita Ct value detected from the isolation of ST from groups A or B and (ii) the Ct value in ST inoculated with Margarita-infected naïve PBMC extract, being ΔCt = Ct_(a)_-Ct _(b)_. The whole protocol was repeated twice, in ST and also in SK6 cells using PBMCs from animals 1 (Group A), 4 and 5 (group B) and cells from the naïve animal (number 1, Group A), collected before Margarita infection.

### 2.9. Sequence analysis

The E2-gene fragment reported by Lowings et al. [44] was amplified by end point RT-PCR [45] in sera, tonsil, lung and spleen from animals 1, 3 (Group A), 4 and 5 (Group B), collected at necropsy. Additionally, the viral inoculums used in the experimental infections (Cat01 and Margarita strains) were evaluated. The amplification products were checked by electrophoresis on 2% agarose gel and were directly cleaned with a Wizard® PCR Preps DNA Purification System (Promega, Madison, Wisconsin, USA). Sequencing reactions were conducted under BigDye^TM^ terminator-cycling conditions using an ABI 3130XL. Forward and reverse sequences obtained from each amplicon were assembled using the Contig Express application in Vector NTI software, version 11 (Invitrogen). The sequences from the E2-gene fragment obtained were aligned to analyse the sequence found in each sample.

## 3. Results

### 3.1. Specificity and sensitivity of Margarita strain real-time TaqMan assay

Of the 20 CSFV RNA strains analysed, the assay detected only the CSFV RNA from the Margarita strain (1.4 genogroup), while the other 19 CSFV RNA extractions were negative (Table 1). This result indicated that the newly developed assay was highly specific for the detection of the CSFV Margarita strain, and there was no cross-reactivity with the other tested CSFV strains from genogroup 2 (including the Cat01 strain). The specificity of the assay was based primarily on mismatches in the probe-binding region but also to some extent on mismatches in primer-binding regions. The sensitivity of the assay was evaluated by testing 10-fold dilutions of the Margarita strain RNA. The analytical sensitivity was estimated to be as high as 0.4 TCID_50_. The assay had a reaction coefficient (R^2^) of 0.994 (data not shown). Positive results were considered for threshold cycle values (Ct) equal to or less than 38. Finally, the presence of Cat01 RNA strain in the sample containing the Margarita strain RNA did not affect the assay sensitivity (Data not shown).

### 3.2. Wild boars persistently infected with CSFV were clinically protected after infection with a CSFV Margarita virulent strain

**Fig 2:**
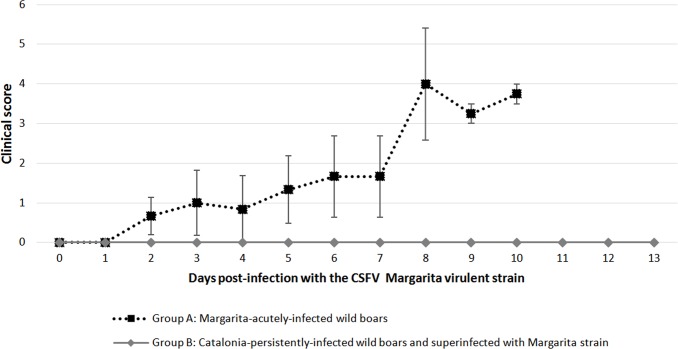
The animals persistently infected with Cat01 were clinically protected after infection with the virulent Margarita strain. Means and standard deviations of the daily individual clinical score values after CSFV virulent Margarita strain infection are represented. Dark grey bars indicate the standard deviation values for group A. The clinical score values are defined in the Materials and Methods section.

Animals persistently infected with the Cat01 strain and inoculated with the virulent Margarita strain (Group B) showed neither clinical signs of disease nor fever at any time throughout the study, maintaining good health status (Fig 2). In contrast, animals from group A, infected with the Margarita strain, presented mild clinical signs at 2 dpi that progressed to moderate within 48–72 h. At 7 dpi, animal 2 showed a clinical score value of 4; however, it was found dead at 8 dpi, with lesions of haemorrhagic diathesis. Animals 1 and 3 progressed to dyspnoea, weight loss, swaying movement of the hindquarters, posterior paresis and high fever until 10 dpi, when euthanasia was performed.

### 3.3. Absence of detectable Margarita strain RNA in CSFV-superinfected wild boars

**Fig 3:**
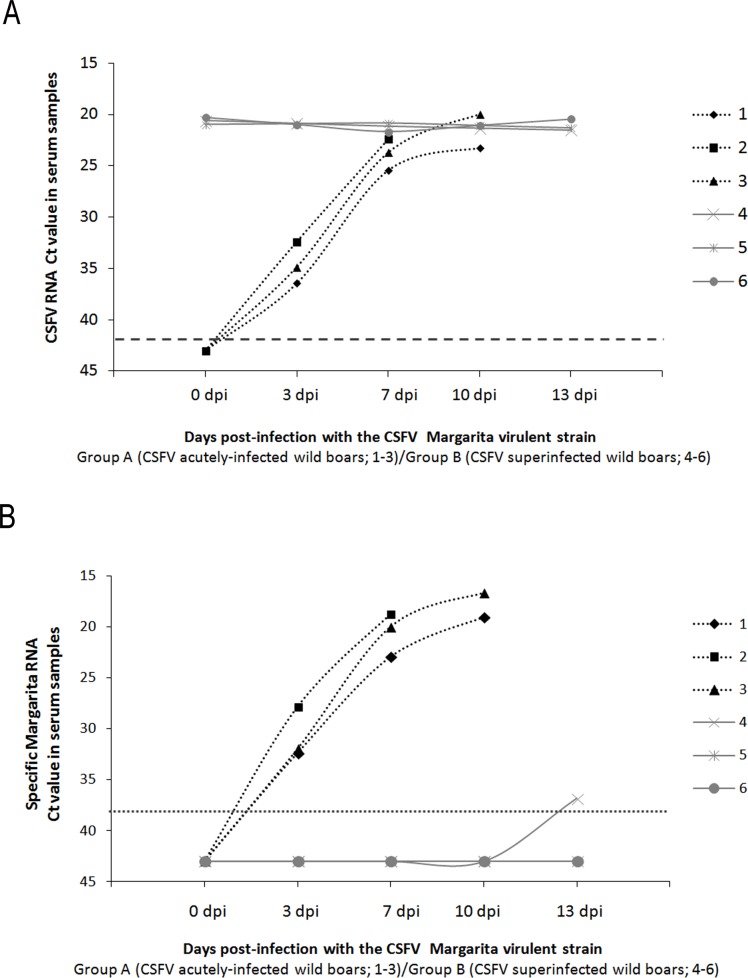
Swine persistently infected with the CSFV Cat01 strain were protected from the typical viraemia generated by the CSFV Margarita strain. (A) Daily detection of CSFV RNA through generic qRT-PCR in sera [39]. The Ct values from group A (CSFV acutely Margarita-infected wild boars; 1–3) and group B (CSFV-superinfected wild boars; 4–6) are represented in black and grey colours, respectively. (B) Daily detection of CSFV RNA Margarita strain through specific qRT-PCR in serum. The Ct mean values from group A (CSFV acutely infected wild boars; 1–3) and group B (CSFV-superinfected wild boars; 4–6) are represented in black and grey colours, respectively. Positive results for the CSFV RNA detection [39] were considered for Ct values equal to or less than 42, indicated with a dashed line. Positive results for the specific CSFV RNA Margarita strain detection were considered for Ct values equal to or less than 38, indicated with a dotted line

Margarita RNA was undetected in the sera from animals in group B, except for animal 4 at 13 dpi with a high Ct value (Ct 36.84), considered a low RNA viral load (36, 39) (Fig 3). Additionally, CSFV Margarita strain RNA could not be detected in any of the nasal or rectal swabs collected from group B (data not shown). Furthermore, in group B, CSFV Margarita RNA was found only in the liver of animal 4 and also in the spleen of animals 4 and 5, with a low RNA viral load. In contrast, all wild boars from group B (CSFV persistently infected with Cat01 strain) maintained during the whole trial a high and constant CSFV RNA load in serum, swabs and organs, when examined by generic CSFV q-RT-PCR (Table 2).

**Table 2:** Swine persistently infected with the CSFV Catalonia strain are protected from CSFV Margarita strain infection in tissue samples.

	Group A (CSFV acutely infected wild boars; 1–3)						Group B (CSFV-superinfected wild boars; 4–6)					
	1		2		3		4		5		6	
Tissues	CSFV RNA ^a^	Margarita RNA ^b^	CSFV RNA	Margarita RNA	CSFV RNA	Margarita RNA	CSFV RNA	Margarita RNA	CSFV RNA	Margarita RNA	CSFV RNA	Margarita RNA
Spleen	18,57	17,70	17,2	17,90	17,09	16,23	21,4	32,91	20,35	36,15	20,85	*Undet*.
Liver	22,3	21,36	21,05	21,94	19,35	19,50	23,96	32,24	24,01	*Undet*.	25,05	*Undet*.
Mes. Ln.^c^	21,78	21,04	21,4	20,32	18,05	16,43	Not det.^g^	Not det.	22,31	*Undet*.	22,76	*Undet*.
Pres. Ln.^d^	19,27	18,12	18,91	18,02	17,88	16,16	22,92	*Undet*.^h^	23,81	Undet.	25,15	*Undet*.
B.M.^e^	19,9	17,92	19,68	16,77	19,49	18,34	19,54	*Undet*.	22,75	*Undet*.	20,72	*Undet*.
M. oblongata^f^	26,44	25,94	26,99	24,86	24,41	25,27	Not det.	Not. Det.	24,4	*Undet*.	24,64	*Undet*.
Urine	31,08	28,49	17,95	18,94	31,76	28,77	20,05	*Undet*.	Not. Det	Not. Det.	21,77	*Undet*.
Lung	23,57	22,70	22,66	21,01	19,14	17,78	20,21	*Undet*.	19,84	*Undet*.	19,97	*Undet*.
Kidney	25,08	23,32	25,24	23,25	21,98	20,57	21,62	*Undet*.	22,55	*Undet*.	21,77	*Undet*.
Thymus	27,46	25,18	23,64	21,31	21,02	19,36	20,5	*Undet*.	21,23	*Undet*.	21,15	*Undet*.
Tonsil	20,33	19,91	21,36	19,06	18,4	16,36	22,87	*Undet*.	25,03	*Undet*.	21,49	*Undet*.

^a^ Ct value detected with the generic CSFV q RT-PCR assay (37).

^b^ Ct value detected with the specific CSFV RNA Margarita strain qRT-PCR assay.

^c^ Mes. Ln = Mesenteric lymph node.

^d^ Pres. Ln. = Prescapular lymph node.

^e^ B.M. = Bone marrow.

^f^ M. oblongata = Medulla oblongata.

^g^ Not Det. = Not determined.

^h^ Undet. = Undetected.

In contrast, both qRT-PCRs (generic and specific for Margarita strain) were positive in organs and samples collected from animals in group A (Table 2). The Ct values were positive by the CSFV generic qRT-PCR [39], in both serum and swab samples, from 3 dpi onwards. Ct values for the specific Margarita assay were similar to those obtained by the CSFV generic qRT-PCR.

### 3.4. Absence of humoral response in terms of E2-specific and neutralising antibodies in CSFV-superinfected animals

To evaluate the induction of CSFV-specific antibodies, serum samples were analysed at different times after CSFV Margarita strain infection. The absence of antibody response, in terms of E2-specific antibodies and neutralising antibody titres, was found in both CSFV acutely and persistently superinfected groups during the entire experiment (Data not shown).

### 3.5. Levels of endogenous IFN-α increased with progression of acute disease but remained undetectable in CSFV-superinfected animals

Previously, it was shown that CSFV PI animals were unable to elicit an innate immune response, in terms of IFN-α production, against a CSFV life-attenuated vaccine [7]. However, we wondered whether superinfection with a CSFV virulent strain would trigger detectable levels of IFN-α in the CSFV-superinfected wild boars (Group B), given that IFN-α has been largely related to disease severity, as a hallmark of CSFV acute infection [34, 46]. In the present work, we observed that progression of disease in group A was correlated with an increase in the levels of endogenous IFN-α after infection, as measured by ELISA, with values that reached more than 240 U/ml in two of three animals at 7 dpi and 10 dpi (data not shown). In contrast, IFN-α was undetectable in all of the serum samples analysed both before (day 0) and after Margarita inoculation of CSFV Catalonia persistently infected pigs (Group B) (data not shown).

### 3.6. CSFV-specific IFN-γ-producing cells were lacking in CSFV-superinfected animals

PBMCs from all of the animals were analysed for virus-specific and non-specific IFN-γ responses by ELISPOT assay at 0 and 13 dpi post-Margarita strain inoculation. Very few IFN-γ-producing cells were found upon CSFV and PHA stimulation of PBMCs from all 3 of the CSFV-superinfected animals (Group B). These results supported our previous results showing that postnatal infection of piglets with CSFV could result in virus persistence due to a lack of B- and T-cell responses (data not shown).

### 3.7. CSFV interference in the PBMCs from CSFV-superinfected hosts

It is well known that white blood cells, including the PBMCs, are targets for CSFV replication [47,48]. Consequently, to examine whether the PBMCs collected from the CSFV-superinfected animals (group B) and the acutely infected animals (group A), were permissive (or not) to CSFV superinfection, we assayed *in vitro* inoculation of such samples, with either Cat01 or Margarita CSFV strains. Similarly, PBMC samples were mock-infected. Additionally, PBMCs from a naïve animal were used as controls. As was expected, CSFV-specific Margarita RNA was detected in the PBMCs from animals developing the CSF acute disease (group A) in both mock and Margarita-infected samples. Furthermore, PBMCs from group B *in vitro* inoculated with Margarita were also positive for CSFV-specific Margarita RNA detection, but with a high Ct value correlated with a lower RNA load (Table 3). Otherwise, PBMCs from group B *in vitro* mock-infected were negative for CSFV-specific Margarita RNA detection (Table 3). Following these findings, to decipher whether the detected RNA load in group B might correspond to RNA traces from the inocula or to the infecting virus, the previously analysed PBMC extracts were inoculated into a ST cell line. Consistently, the detected RNA load notably increased in ST after inoculation with the extract from Margarita *in vitro* inoculated-naïve PBMCs; the obtained 7.76Δ Ct positive value confirmed the infectivity of the virus recovered from the PBMC samples. In contrast, Margarita RNA in group B *in vitro* mock-infected PBMCs remained undetectable even after ST inoculation. Furthermore, Margarita RNA load detection in group B *in vitro* Margarita-infected PBMC samples decreased after inoculation of ST cells, corresponding to higher Ct values than those previously detected directly from PBMC extracts. Remarkably, an 11.6 ΔCt value was found in the ST cells with Margarita *in vitro* inoculated PBMCs from group B, relative to the value obtained in the ST cell extracts from Margarita-inoculated naïve PBMCs (Table 3). The whole protocol was repeated twice for animals 1 (group A), 4 and 5 (group B) in both SK6 and ST cells, supporting the results with similar Ct values (data not shown). Similarly, the cells’ positive infection was confirmed by PLA testing, although this test cannot differentiate between Cat01 and Margarita CSFV strains.

**Table 3:** CSFV interference in the PBMCs from superinfected hosts.

	Experimental groups					
	Naive		CSFV acutely infected wild boar 1 (10 dpi; group A)		CSFV-superinfected wild boar 5 (13 dpi; group B)	
	CSFV Ct ^a^	Specific Margarita Ct^b^	CSFV Ct	Specific Margarita Ct	CSFV Ct	Specific Margarita Ct
**PBMC extracts**						
Mock-infected PBMC	*Undetected*	*Undetected*	24.99	23.10	26.38	*Undetected*
CSFV Catalonia-infected PBMCs (MOI 0.1)	34.90	*Undetected*	25.86	23.61	25.60	*Undetected*
CSFV Margarita-infected PBMCs (MOI 0.1)	32.50	31.47	25.06	22.91	25.14	31.05
**ST cell extracts**						
Mock	*Undetected*	*Undetected*	29.70	27.81	25.57	*Undetected*
CSFV Catalonia (MOI 0.1)	25.93	*Undetected*	29.37	27.95	28.67	*Undetected*
CSFV Margarita (MOI 0.1)	26.80	23.71	28.14	25.83	28.92	35.31
**CSFV Margarita Δ CT value determination**						
PBMC extracts—ST cell extracts		7.76^c^		-2.92^c^		-4.26^c^
ST cell extracts—ST cell extracts from naïve animals				2.12^c^		11.6^c^

^a^ Ct value detected with the generic CSFV qRT-PCR assay (37).

^b^Ct value detected with the specific CSFV RNA Margarita strain q RT-PCR assay.

^c^Δ Ct value.

### 3.8. Sequence analysis could not detect CSFV Margarita RNA in tissues from CSFV-superinfected animals

**Fig 4:**
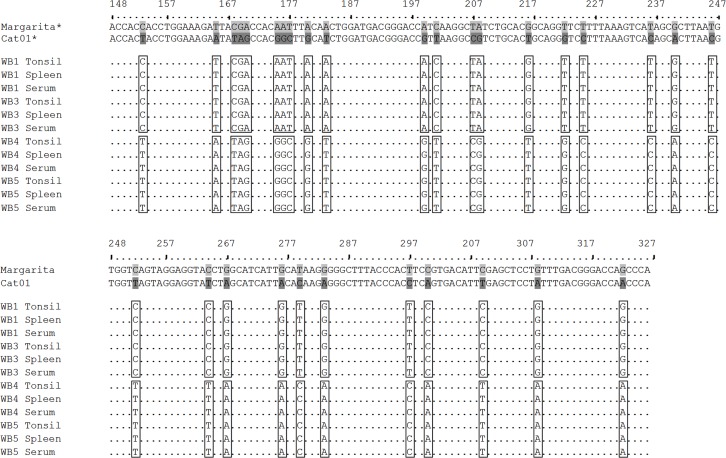
Sequence analysis of the partial E2 sequence does not detect the CSFV Margarita RNA in the tissues from superinfected animals. The Margarita and Cat01 viral strain sequences used as viral inocula in the animal infection experiments were considered as references. Sequences from sera, tonsil and spleen samples from group A (CSFV acutely infected wild boars; 1, 3) and group B (CSF-superinfected wild boars; 4, 5) are shown. Differences in the nucleotide sequences between the CSFV Margarita and Catalonia 01 strains are shown in grey and dark grey, respectively.

To detect the presence of CSFV RNA of both viral strains (Cat01 and/or Margarita) in the sera, tonsil, and spleen of animals 1 and 3 (Group A) and 4 and 5 (Group B), the E2-gene fragment reported by Lowings et al. [44] as a phylogenetic marker was amplified by end point RT-PCR [45]. In all of the samples analysed from animals that developed the CSF acute form (Group A), the sequence corresponding to the Margarita strain (AJ704817) used as the inoculum was detected. Furthermore, the samples analysed from superinfected animals (Group B: CSFV Catalonia 01 persistently infected inoculated with CSFV Margarita strain) only showed the sequence corresponding to the Cat01 strain [30] (Fig 4).

## 4. Discussion

Despite its significance, the mechanisms of mutual exclusion by viral variants are far from being completely understood, and the actual knowledge is basically derived from studies at the cellular level in established cell lines [14, 16, 19, 49]. Very few reports have demonstrated the phenomenon of SIE at the organism level, and, to our knowledge, these models have been limited to plant viruses, West Nile virus (WNV) in mosquitoes, and Peking duck hepatitis B virus (DHBV) [50–52]. In addition, it has not yet been demonstrated in a mammalian host at the systemic level.

Previous works have reported the capability of CSFV to generate postnatally persistent infection in both domestic pigs and wild boars [5, 6]. Subsequently, it was also shown that postnatally persistently infected pigs were unable to elicit a specific immune response to a CSFV live attenuated vaccine and that the viral vaccine RNA was undetectable in any of the samples analysed [7]. Against this background, we assessed the capacity of CSFV to generate SIE in CSFV persistently infected swine. For that purpose, CSFV persistently infected wild boars were inoculated with a CSFV strain that induce acute disease with a higher replication rate [29, 36].

Because pestiviruses are immunologically and genetically closely related, accurate serological characterisation of CSFV isolates is impeded by the extensive cross-reactions observed among Pestivirus members and the limited availability of MAbs capable of differentiating among different CSFV isolates [27, 45, 53]. To differentiate the CSFV Margarita strain RNA from the CSFV Cat01 strain RNA in the samples from the present study, a specific qRT-PCR for Margarita strain RNA detection was developed. Thus, alongside the model of infection with the Margarita strain, the qRT-PCR assay developed allowed for clear discernment of whether there was actually a blockage that prevented susceptibility to infection by the second virus in both the absence of clinical signs and the absence of molecular detection of the superinfecting virus.

Notwithstanding the high infection rate of the Cat01 strain in persistently infected animals from group B (primary virus infection), good health status was maintained after inoculation with the Margarita CSFV virulent strain (secondary infection) in the absence of viral detection in sera throughout the study, except in one animal at 13 dpi with a low Margarita strain RNA load (animal 4). Despite the important role that neutralising antibodies play in CSFV protection [29, 54], complete absence of neutralising antibodies response was found after Margarita strain infection in these animals. Similarly, absence of an IFN-γ-producing cell response against CSFV or PHA was also observed. Considering the role played by IFN-γ in the control of CSFV infection [34, 55] and the lack of responsiveness to IFN-γ-producing cells after PHA stimulation, the CSFV-superinfected animals maintained a immunosuppression state similar to that previously described in postnatal persistent infection [5, 6]. Previous work has proved how the failure to induce optimal levels of the humoral and cellular responses after CSFV infection promoted the spread of the virus and its relationship with disease progression [29, 54]. In this regard, the implications of the cellular and neutralising antibody response in clinical protection against the acute form in the CSFV-superinfected animals from this study are excluded.

Furthermore, no superinfecting virus excretion was detected in any of the animals from Group B, whilst the high viral load generated by the strain that induced the persistent infection (Cat01 strain or primary infection) was maintained until the end of the trial, supporting our previous results [7]. In contrast, the CSFV Margarita strain generated the acute form of the disease in animals from group A, with high Margarita RNA loads in all of the samples analysed. In addition, the failure of the humoral response in the pigs that developed acute CSF was previously described [29].

In addition to the adaptive immune response, the innate immune response to the virus, as measured by type I IFN-α in the serum, also seemed to be impaired, in terms of IFN-α detection because IFN-α values were undetectable in the sera from postnatally persistently infected wild boars after CSFV Margarita strain inoculation. At the same time, the progression of the acute disease in group A was correlated with an increase in levels of endogenous IFN-α, as has been previously described [29, 46, 56, 57]. The absence of an IFN-α response in the Cat01 persistently infected animals after Margarita strain inoculation (secondary infection) probably was due to the almost complete lack of Margarita strain replication in these animals. Otherwise, specific CSFV-blockade phenomena for IFN-α might be occurring. Efficient viral strategies to escape the type I IFN-induced antiviral mechanisms have been described within Pestivirus. In this regard, the viral RNA triggers IFN synthesis, and the viral RNase E^rns^ inhibits IFN expression induced by extracellular viral RNA [58]. In addition, the viral protein N^pro^ suppresses type I IFN (IFN-α/β) induction by mediating proteasomal degradation of IFN regulatory factor 3 (IRF-3) [58–60]. For instance, in persistent infection, BVDV maintains “self-tolerance” by avoiding the induction of IFN, without compromising the IFN action against unrelated viruses (“nonself”) [58]. In the case of CSFV-infected pigs, it has been recently demonstrated that functional N^pro^ significantly reduced local IFN-α mRNA expression responses at local sites of virus replication [61]. These highly selective “self” models of evasion of the interferon defence system might be key elements in the success of persistent infections and could promote, in addition, the generation of SIE phenomena.

Previous reports have suggested that the availability of mammalian models for SIE *in vivo* is hampered by the interferon response generated against the infecting virus in these species [11, 24]. It is noteworthy that CSFV postnatally persistently infected swine have shown an immunosuppression state comprising a reduction in interferon responses (Types I and II) [5–7]. This immunological status might promote the maintenance of a high and constant CSFV load, as already described, preventing second viral entry [5]. Nevertheless, further studies would be needed to clarify the molecular mechanisms involved in this phenomenon.

At 13 dpi, low levels of Margarita RNA were detected only in some collected tissues from persistently and superinfected wild boars (Group B), principally in animal 4, in which Margarita RNA was detected from the spleen and liver, as well as in the serum. However, the level of Margarita RNA detection was approximately fifteen times less than the acutely infected animals from group A (Table 2). The Margarita RNA levels found in the superinfected animals might be correlated with the low Margarita strain viral loads in some macrophages in these tissues [62].

In contrast, despite PBMCs being a well-known target for CSFV viral replication [62], after *in vitro* assay, the presence of CSFV Margarita RNA could not be detected in either the PBMCs or ST cell extracts from Group B. Additionally, the *in vitro* superinfection of isolated PBMCs failed when they were derived from persistently infected piglets but were clearly positive for assays with cells from naïve animals, as demonstrated by means of calculated ΔCt values, supporting that PBMCs from persistently infected animals were substantially protected from superinfection after *in vitro* inoculation with the Margarita virus strain. These results suggest that SIE still occurs at the tissue level (Table 3). In contrast, the Margarita strain RNA could not be detected after the sequence analyses of the samples from persistently infected Margarita-inoculated animals (Group B) nor even in the tonsil, one of the main targets for CSFV replication [3, 63]. Nevertheless, next-generation sequence analyses would be of great interest to analyse these samples in detail, emphasising the spleen and liver tissues that were also positive for RNA Margarita strain detection after superinfection.

Altogether, although it is a very complex mechanism, if compared with the acutely infected group A, these results showed that a phenomenon of CSFV SIE occurred at the systemic level. Nevertheless, the colonisation of a multi-cellular host is a complex process during which the viral load can dramatically change in different organs and at different stages of the infection, and not all of the potential target cells are infected in persistently infected animals despite the high viral load generated by the Cat01 CSFV strain in persistently infected animals [5, 6]. Illustrative examples include some of the works performed to demonstrate SIE at the cellular level because some cells uninfected by one viral primary infection are subsequently infected by the second viral infection [11, 50]. In contrast, the implications of other mechanisms in the host cannot be excluded, and it remains unclear whether the observed phenomenon is really due to a blockage at the level of infection of cells. This was precisely in the case of a citrus tristeza virus (CTV) SIE model, wherein a CTV protein (p33) was required to mediate SIE at the organism level but that did not appear to be implicated in exclusion at the cellular level [50].

Overall, our results suggested efficient suppression of viral superinfection in a mammalian host, especially in the absence of IFN-α, indicating a lack of innate immune mechanisms. Considering the role of this phenomenon from an evolutionary standpoint, their implications within an epidemic situation might be relevant to the evolution and phylogeny of CSFV. Although this phenomenon must be studied in greater depth, the possible outcome for the generation of new CSFV strains circulating in an endemic situation and the impact on disease control, including vaccination with live attenuated vaccines, cannot be underrated.
